# The PI3-Kinase Delta Inhibitor Idelalisib (GS-1101) Targets Integrin-Mediated Adhesion of Chronic Lymphocytic Leukemia (CLL) Cell to Endothelial and Marrow Stromal Cells

**DOI:** 10.1371/journal.pone.0083830

**Published:** 2013-12-23

**Authors:** Stefania Fiorcari, Wells S. Brown, Bradley W. McIntyre, Zeev Estrov, Rossana Maffei, Susan O’Brien, Mariela Sivina, Julia Hoellenriegel, William G. Wierda, Michael J. Keating, Wei Ding, Neil E. Kay, Brian J. Lannutti, Roberto Marasca, Jan A. Burger

**Affiliations:** 1 Department of Leukemia, The University of Texas MD Anderson Cancer Center, Houston, Texas, United States of America; 2 Department of Immunology, The University of Texas MD Anderson Cancer Center, Houston, Texas, United States of America; 3 Division of Hematology, Department of Internal Medicine, Mayo Clinic, Rochester, Minnesota, United States of America; 4 Gilead Sciences, Inc. Seattle, Washington, United States of America; 5 Hematology Unit, Department of Medical and Surgical Sciences, University of Modena and Reggio Emilia, Modena, Italy; University of Manitoba, Canada

## Abstract

CLL cell trafficking between blood and tissue compartments is an integral part of the disease process. Idelalisib, a phosphoinositide 3-kinase delta (PI3Kδ) inhibitor causes rapid lymph node shrinkage, along with an increase in lymphocytosis, prior to inducing objective responses in CLL patients. This characteristic activity presumably is due to CLL cell redistribution from tissues into the blood, but the underlying mechanisms are not fully understood. We therefore analyzed idelalisib effects on CLL cell adhesion to endothelial and bone marrow stromal cells (EC, BMSC). We found that idelalisib inhibited CLL cell adhesion to EC and BMSC under static and shear flow conditions. TNFα-induced VCAM-1 (CD106) expression in supporting layers increased CLL cell adhesion and accentuated the inhibitory effect of idelalisib. Co-culture with EC and BMSC also protected CLL from undergoing apoptosis, and this EC- and BMSC-mediated protection was antagonized by idelalisib. Furthermore, we demonstrate that CLL cell adhesion to EC and VLA-4 (CD49d) resulted in the phosphorylation of Akt, which was sensitive to inhibition by idelalisib. These findings demonstrate that idelalisib interferes with integrin-mediated CLL cell adhesion to EC and BMSC, providing a novel mechanism to explain idelalisib-induced redistribution of CLL cells from tissues into the blood.

## Introduction

Chronic lymphocytic leukemia (CLL) is characterized by the expansion of monoclonal CD5^+^/CD23^+^ B lymphocytes in the peripheral blood, bone marrow, and secondary lymphatic tissues [1]. CLL B cells accumulate in vivo, but undergo spontaneous apoptosis in vitro, unless they are co-cultured with supportive stromal cells. This suggests that in vivo CLL cells interact with accessory cells in tissue microenvironments which provide growth- and survival-signals [2]. Previous studies demonstrated that co-culture with different types of stromal cells, such as monocyte-derived nurselike cells (NLC) [3], bone marrow stromal cells (BMSC) [4,5] and endothelial cells (EC) [6,7] promotes CLL cell survival and protects from spontaneous or drug-induced apoptosis. It is also well recognized that CLL cell growth occurs in characteristic lymphatic tissue areas called proliferation centers or pseudofollicles [8], where leukemia cell proliferation accounts for a daily turnover of up to 1 to 2% of the entire CLL cell clone [9]. Hence, based on *in vitro* and in vivo studies it is now recognized that crosstalk between CLL cells and the tissue microenvironment plays a critical role in regard to the expansion of the CLL clone [10]. Concurrent with these new insights into CLL disease pathogenesis, novel kinase inhibitors interfering with the proactive role of the microenvironment, particularly with B cell receptor (BCR) signaling are under development in CLL, and demonstrate encouraging clinical activity in early stage clinical trials [11–13]. 

Idelalisib, previously called GS-1101 or CAL-101, is a potent and selective inhibitor of the PI3Kδ isoform delta (PI3Kδ) [14]. Idelalisib induces apoptosis in B cell lines and primary B cells from patients with different B-cell malignancies, including CLL [15,16], diffuse large B-cell lymphoma [14], multiple myeloma [17] and Hodgkin lymphoma [18]. Several lines of evidence demonstrate that idelalisib interferes with the crosstalk between CLL cells and their microenvironment. Idelalisib inhibits CLL cell signaling pathways in response to CD40L, BAFF, TNF-α, fibronectin and stromal cells [19]. Furthermore, idelalisib affects CLL cells migration beneath BMSC, chemotaxis towards the chemokines CXCL12 and CXCL13, and disrupts BCR signaling and BCR-induced secretion of the CLL cell-derived chemokines CCL3 and CCL4 [16]. 

Inhibition of CLL cell migration alone cannot fully explain idelalisib-induced redistribution of CLL cell from tissues into the blood, given that normal lymphocyte trafficking and homing require intimate cooperation between adhesion molecules and chemokine receptors [20]. Normal blood lymphocytes interact transiently and reversibly with endothelial cells through membrane receptors defined as selectins and integrins in a process called rolling. Chemokines on the luminal endothelial surface then activate chemokine receptors on the rolling cells, which triggers integrin activation [20], resulting in cell arrest, firm adhesion, and transendothelial migration into tissues, where chemokine gradients direct lymphocyte localization and retention [21]. VLA-4 integrin plays a critical role in leukocytes trafficking, adhesion and survival through the binding with VCAM-1 or fibronectin [22]. VLA-4 is variably expressed by CLL patients and predicts disease progression. CLL patients with higher VLA-4 expression are characterized by more rapid disease progression when compared to patients with low expression [23,24]. Moreover, VLA-4 expression increases the ability of CLL cells to access protective niches [25]. Given the critical role of integrin-mediated adhesion for normal lymphocytes trafficking between blood and secondary lymphoid tissues [26,27] and the important role of VLA-4 in CLL pathogenesis, we hypothesized that idelalisib interferes with integrin function and signaling. We therefore examined the mechanism of idelalisib modulation of integrin-mediated CLL cell adhesion and integrin signaling events.

## Materials and Methods

### CLL cell purification, reagents

Written inform consent was obtained in accordance with the Declaration of Helsinki on protocols approved by the University of Texas MD Anderson Cancer Center Institutional Review Board (IRB), before peripheral blood samples were collected from untreated CLL patients fulfilling clinical and immunophenotypic criteria for CLL. Peripheral blood mononuclear cells (PBMCs) were isolated by density gradient centrifugation over Ficoll-Pacque (GE Healthcare, Waukesha, WiWi, USA) and were used fresh or cryopreserved in fetal bovine serum (FBS, BD Biosciences, San Diego, CA, USA) plus 10% dimethylsulfoxide (Sigma-Aldrich, St Louis, MO, USA). Purity of CD19^+^ CLL cells was assessed to be > 90% by flow cytometry. Idelalisib was provided by Gilead Sciences (Seattle, WA, USA), dissolved in DMSO at 10mM, and stored at -20°C until use.

### Cell culture

The murine stromal cell lines KUSA-H1 and 9-15c, both derived from bone marrow of a C3H/He mouse, were purchased from the RIKEN Cell Bank (Ibaraki, Japan) and maintained in RPMI 1640 medium supplemented with 2.05 mM L-glutamine (Hyclone, Logan, UT), 10% FBS (SACF Biosciences) and penicillin-streptomycin (Cellgro). The human mesenchymal cell line StromaNKtert, derived from bone marrow and immortalized by human telomerase reverse transcriptase (hTERT)[28] containing also exogene MFG-tst-IRES-neo was purchased from RIKEN and maintained in minimum essential Medium Eagle with Earl salts and L-glutamine (α-MEM; HyClone) supplemented with 12.5% FBS (SACF Biosciences, Lenexa, KS), 12.5% human serum (Cellgro), 1μM hydrocortisone (Sigma-Aldrich, St. Louis, MO) and 100μM 2-mercaptoethanol (Sigma-Aldrich). Primary human MSCs, isolated from bone marrow of CLL patients, were isolated and expanded as previously described [29]. Human umbilical vein endothelial cells (HUVEC, Cascade Biologics, Portland OR) pooled from multiple donors, were cultured in M200 PRF medium supplemented with Low Serum Growth Supplement kit (Cascade Biologics) with a final concentration of 2% FBS, 1μg/ml hydrocortisone, 10ng/ml human epidermal growth factor, 3 ng/mL basic fibroblast growth factor and 10 μg/mL heparin. The human microvascular endothelial cell line HMEC-1 was purchased from the Centers for Disease Control and Prevention (CDC, Atlanta, GA) and was maintained in MCDB131 medium (Invitrogen, Carlsbad, CA) supplemented with 15% FBS (SACF Biosciences), 10 mM/L L-glutamine (Hyclone), 10ng/mL Epidermal Growth Factor (Becton-Dickinson) and 1μg/mL hydrocortisone (Sigma-Aldrich). The murine vascular endothelial cell line UV-2, transformed by ultraviolet, was purchased from RIKEN and maintained in DMEM medium supplemented with 10% FBS (SACF Biosciences), 2.05 mM L-glutamine (Hyclone) and penicillin-streptomycin (Cellgro). RAMOS cells (ATCC, LGC Standards, UK) were cultured in RPMI 1640 supplemented with 2.05 mM L- glutamine (Hyclone, Logan, UT), 10% FBS (SACF Biosciences) and penicillin-streptomycin (Cellgro). 

### Static cell adhesion assay

Two different EC (HUVEC and HMEC-1) and two different BMSC (9-15c and CLL-MSC) were seeded for 24 hours onto 24-well plate at a concentration of 0.8x10^5^ cells/ml in the appropriate cell culture medium and then stimulated for 24 hours with 10ng/mL TNFα (R&D Systems, Minneapolis, MN)[30]. After 1 hour incubation at 37°C and 5% CO_2_ in complete medium, CLL cells were added onto the confluent endothelial and stromal cell layers to a final concentration of 5x10^6^/well in presence or absence of 5 μM idelalisib, and the plates were incubated at 37°C for 4 hours. After incubation the cells that were not adhered to the EC and BMSC cell layers were removed by washing the wells with RPMI 1640 medium. The complete removal of non-adherent cells and the integrity of the cell layer was assessed by phase contrast microscopy and documented photographically. The EC and BMSC layers containing the adherent CLL cells then were detached by incubation for 1 minute with trypsin/EDTA solution pre-warmed at 37°C (Invitrogen), followed by neutralization of the trypsin with 1 ml of RPMI/10% FBS. Cells then were washed and suspended in a final volume of 0.6 ml medium for counting by flow cytometry (FACS Calibur, BD Biosciences) for 20 seconds at 60μl/min in triplicates. A lymphocyte gate was set using the different relative size and granularity (forward scatter and side scatter) to exclude endothelial and stromal cells from the count, as described before [31]. The number of adherent cells under each condition was expressed as percentage of the control for each experiment.

### Parallel plate flow detachment assay

Slides were coated with HUVEC in culture media for 24 hours and then stimulated with TNFα (10ng/ml) (R&D systems) in fresh medium for 24 hours. After 1 hour incubation in complete medium at 37°C in 5% CO_2_, 5x10^6^ CLL cells, treated with 5μM idelalisib for 1 hour at 37°C and untreated controls, were injected into the flow chamber and allowed to settle on the slides for 20 minutes. For cell adhesion to VCAM-1 (R&D Biosystems), slides were coated with 10 μg/ml VCAM-1 or ovalbumine (Sigma-Aldrich) overnight at 4°C. The slides were washed with PBS and blocked with 2% ovalbumine before addition of Ramos. 5x10^6^ Ramos cells were either treated or not treated with 5 μM idelalisib for 1 hour at 37°C and allowed to adhere to VCAM-1 slides for 20 minutes. Using a computer controlled syringe pump (Harvard Apparatus Holliston, MA), an increasing linear gradient of shear flow was applied over the adherent cells for 300 seconds and the number of cells remaining adhered was recorded by digital microscopy. Shear stress calculations were determined every 50 seconds where the shear stress in dynes/cm^2^ was defined as 6μQ/wh^2^ where μ is the viscosity of the medium (0.007), Q is the flow rate in cm^3^/s, w is the width of the chamber (0.3175 cm) and h is the height of the chamber (0.0254 cm). All the samples were assayed in triplicates.

### VCAM-1 and VLA-4 flow-cytometry staining

HUVEC and HMEC-1 were grown in the appropriate cell culture medium until confluence and then stimulated with TNFα (R&D Systems) at a concentration of 10ng/ml for 24 hours and then detached with 5mmol/L EDTA/EGTA in Dulbecco’s PBS (HyClone), pelleted and washed with RPMI 1640 + 0.5% bovine serum albumin (BSA). Then cells were stained with PE-conjugated VCAM-1 antibodies (BD Biosciences) or isotype-matched control antibodies (mouse IgG1-PE; BD Biosciences) and incubated for 20 minutes at 4°C. VLA-4 expression in CLL cells was detected in 10 CLL samples using anti-CD19 APC and anti VLA-4 PE (BD Biosciences) or isotype control. Samples were washed with FACS Buffer and resuspended in 400 μl for assessment on a FACS Calibur (BD Biosciences).

### CLL viability in EC and BMSC co-cultures

For co-culture experiments two different EC (HUVEC, UV-2) and 2 different BMSC (NKtert, KUSA-H1) were seeded the day before the experiment onto a 24-well plate at a concentration of 5x10^4^ cells/well and incubated at 37°C in 5% CO_2_ in the corresponding medium. After confirming the confluence of the cell layer by phase contrast microscopy, CLL cells were added onto the cell layer at a final concentration of 2x10^6^/ml. As control, CLL cells were cultured in medium suspension at a concentration of 2x10^6^/ml. To evaluate the effect of idelalisib on CLL cells viability, co-cultures were treated with 5μM idelalisib. At the indicated time-points, CLL cells were collected and tested for cell viability. Determination of CLL cell viability in the presence or absence of idelalisib was assessed with the analysis of mitochondrial transmembrane potential by 3,3’ dihexyloxacarbocyanine iodide (DiOC6; Molecular Probes, Invitrogen) and cell membrane permeability to Propidium Iodide (PI; Sigma- Aldrich)[3]. CLL cells viability was determined at baseline and after 24, 48 and 72 hours.

### Detection of phospho-proteins by flow cytometry

Phospho-protein expression in CLL cells was detected after 1 hour and 24 hours of co-culture with HMEC-1 and after 1 hour of stimulation with a monoclonal antibody for α_4_β_1_ integrin (19H8) [32] with antibody crosslinking using F(ab’)2 fragments of goat anti-mouse IgG (Invitrogen) in presence or absence of 5μM idelalisib. The following antibodies were used for detection of activated phospho-proteins: anti-pAkt T308 AlexaFluor 488 (Cell Signaling Tecnology) and anti-CD19 PE-cy5 (BD Biosciences) or an isotype-matched control antibody (rabbit IgG-AlexaFluor 488, Cell Signaling Technology). 1x10^6^ CLL cells were suspended in PBS containing 4% paraformaldehyde to block stimulation reaction. After 10 minutes incubation, cells were washed once in cold PBS and stored overnight. Then cells were washed and resuspended in PBS containing 1% bovine serum albumin and incubated with antibodies for 1 hour at room temperature. Samples were washed with PBS + 1% BSA and resuspended in 350 μl PBS + 1% BSA for assessment on a FACS Calibur (BD Biosciences).

### Immunoblotting

CLL cells were starved in RPMI + 0.5% BSA for 2 hours at 37°C then cultured in suspension or co-cultured with HMEC-1 for 1 hour and 24 hours at 37°C. Cells were washed once with cold PBS and lysed ice for 30 minutes with lysis buffer containing 25 mM HEPES, 300 mM NaCl, 1.5 mM MgCl2, 0.5% sodium deoxycholate, 20 mM glycerophosohate, 1% Triton X-100, 0.1% SDS, 0.2 mM EDTA, 0.5 mM dithiothreitol, 1 mM sodium orthovanadate, and protease inhibitor. Cells were pelleted with centrifugation at 14.000 rpm for 15 minutes at 4°C and supernatant was stored at -80°C. Protein content was determined using the detergent compatible (DC) protein assay kit, according to manufacturer’s instructions (Bio-Rad Laboratories, Hercules, CA). Aliquots (50 µg) of total cell protein were boiled with Laemmli sample buffer and loaded onto 4% to 12% SDS-polyacrylamide gradient gels and transferred to nitrocellulose membranes (GE, Osmonics Labstore, Minnetonka, MN). Membranes were blocked for 1 hour in PBS-Tween containing 5% nonfat dried milk and incubated with primary antibodies either overnight or for 3 hours followed by species-specific horseradish peroxidase (HRP)-conjugated secondary antibody (diluted 1:10000) for 1 hour. The blots were visualized by enhanced chemiluminescence according to the manufacturer’s instructions (Pierce Biotechnology, Rockford, IL) and normalized to the actin levels in each extract. Membranes were probed at 4°C with the following primary antibodies: anti-total AKT, phospho-AKT (clones T308 and S473) (Cell Signaling, Danvers, MA), and β-Actin (Sigma- Aldrich). Immunoreactive bands were visualized using peroxidase-conjugated secondary antibodies (GE Healthcare) and enhanced chemiluminescence detection system (Pierce Biotechnology). 

### Data analysis and statistics

Results are shown as mean ± standard error mean (SEM) of at least 3 experiments. Analyses were performed using GraphPad Prism 4 Software for Macintosh (GraphPad software Inc., La Jolla, CA, USA). Student paired or unpaired T tests were used for statistical comparison. The Spearman test was used for estimating correlations between quantitative parameter. A P value ≤ 0.05 was considered statistically significant. Flow cytometry data were analyzed using FlowJo version 8.8.7 software (Treestar).

## Results

### Idelalisib inhibits CLL cell adhesion to EC and BMSC

**Figure 1:**
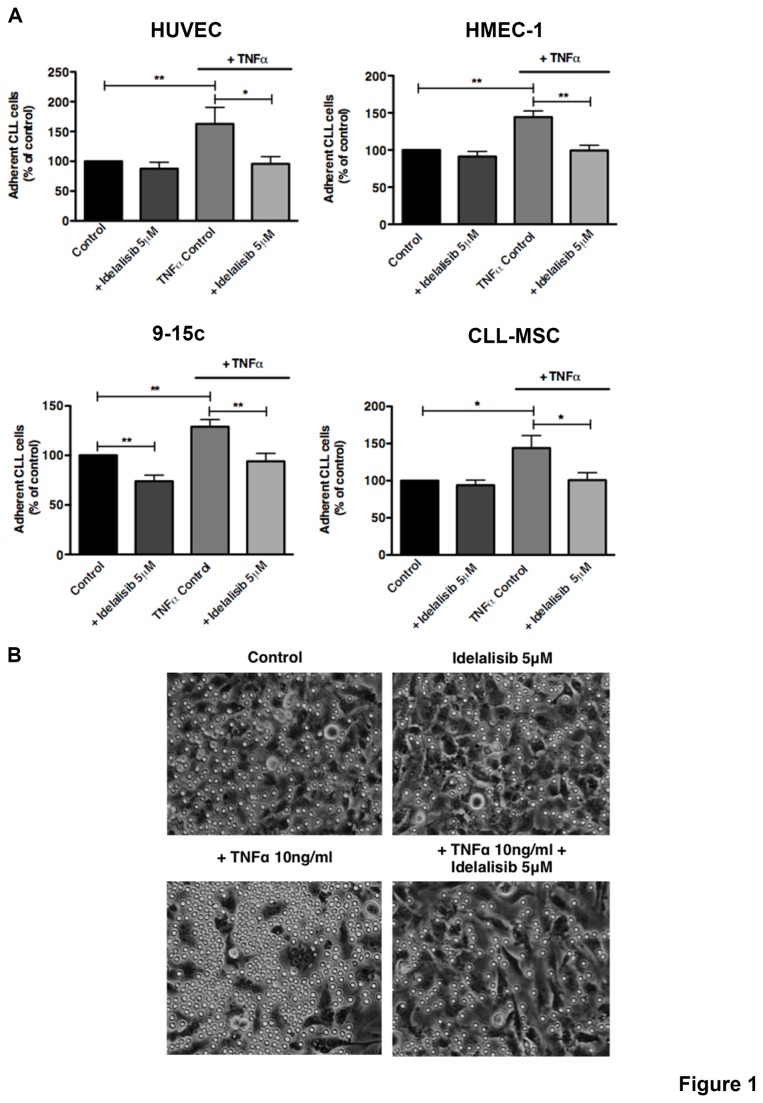
Idelalisib inhibits CLL cell adhesion to TNFα-stimulated EC and BMSC. CLL cells were allowed to adhere to EC (HUVEC or HMEC-1) or BMSC (9-15c or CLL-MSC). EC and BMSC were either unstimulated (control) or stimulated with TNFα (10ng/ml). The bar diagrams represent the mean relative adhesion (±SEM; n=8) of CLL cells in the presence or absence of idelalisib to EC and BMSC compared with the control. TNFα treatment increases the adhesion of CLL cells to EC and BMSC and in presence of idelalisib is significantly inhibited (*p<0.05; **p<0.01). (B) Representative phase contrast photomicrographs demonstrating CLL cell adhesion to HMEC-1 either treated or untreated (control) with TNFα. Treatment with TNFα increased the number of adherent CLL cells to HMEC-1 in comparison to the control (on the bottom left); in presence of idelalisib the number is reduced both in presence of TNFα treatment.

Primary CLL cells displayed a significant increased adhesion to both EC and BMSC stimulated with TNFα when compared to unstimulated controls (Figure 1A). For example the mean relative number (±SEM) of CLL cells adherent to TNFα-treated HMEC-1 increased to 144.3% ± 8.3% (p<0.01, n=8) and to 143.9% ± 16.9% on TNFα-treated CLL-MSC respectively (p<0.05, n=8) when compared to respective untreated controls (Figure 1A). Next, we found that CLL cell adhesion to TNFα-stimulated EC and BMSC was inhibited by idelalisib. The mean relative adhesion (±SEM) of CLL cells to EC was significantly reduced from 162.8% (±27.7%) to 95.7% (±12.1%) using HUVEC, and from 144.3% (±8.3%) to 99.4% (±7%) using HMEC-1 cells. CLL cell adhesion to TNFα-stimulated BMSC was significantly reduced from 128.9% (±7.3%) to 94.1% (±7.9%) using 9-15c cells, and from 143.9% (±16.9%) to 100.7% (±10.1%) using CLL-MSC (Figure 1A). Idelalisib also significantly reduced CLL cell adhesion to unstimulated 9-15c BMSC, but this idelalisib-induced reduction in adhesion of CLL cells to other unstimulated EC or BMSC layers was minor and did not reach statistical significance. CLL cell adhesion was reduced by idelalisib to 91.2% (±6.8%, ns, n=8) on HMEC-1, to 87.5% (±11%, ns, n=8) on HUVEC, to 73.9% (±6.2%, p<0.01, n=8) on 9-15c, and to 93.9% (±6.9, ns, n=8) using CLL-MSC. Phase-contrast photomicrographs document increased numbers of adherent CLL cells after TNFα-treatment and reduced numbers of adherent CLL cells after treatment with idelalisib (Figure 1B). Notably, CLL cells that adhere to TNFα-treated cell layers appear larger in size, presumably because of spreading during the process of adhesion, a well-recognized process that is related to rearrangement of the cytoskeleton [33].

### Idelalisib interferes with cell adhesion to activated endothelium and VCAM-1 under shear flow conditions

**Figure 2:**
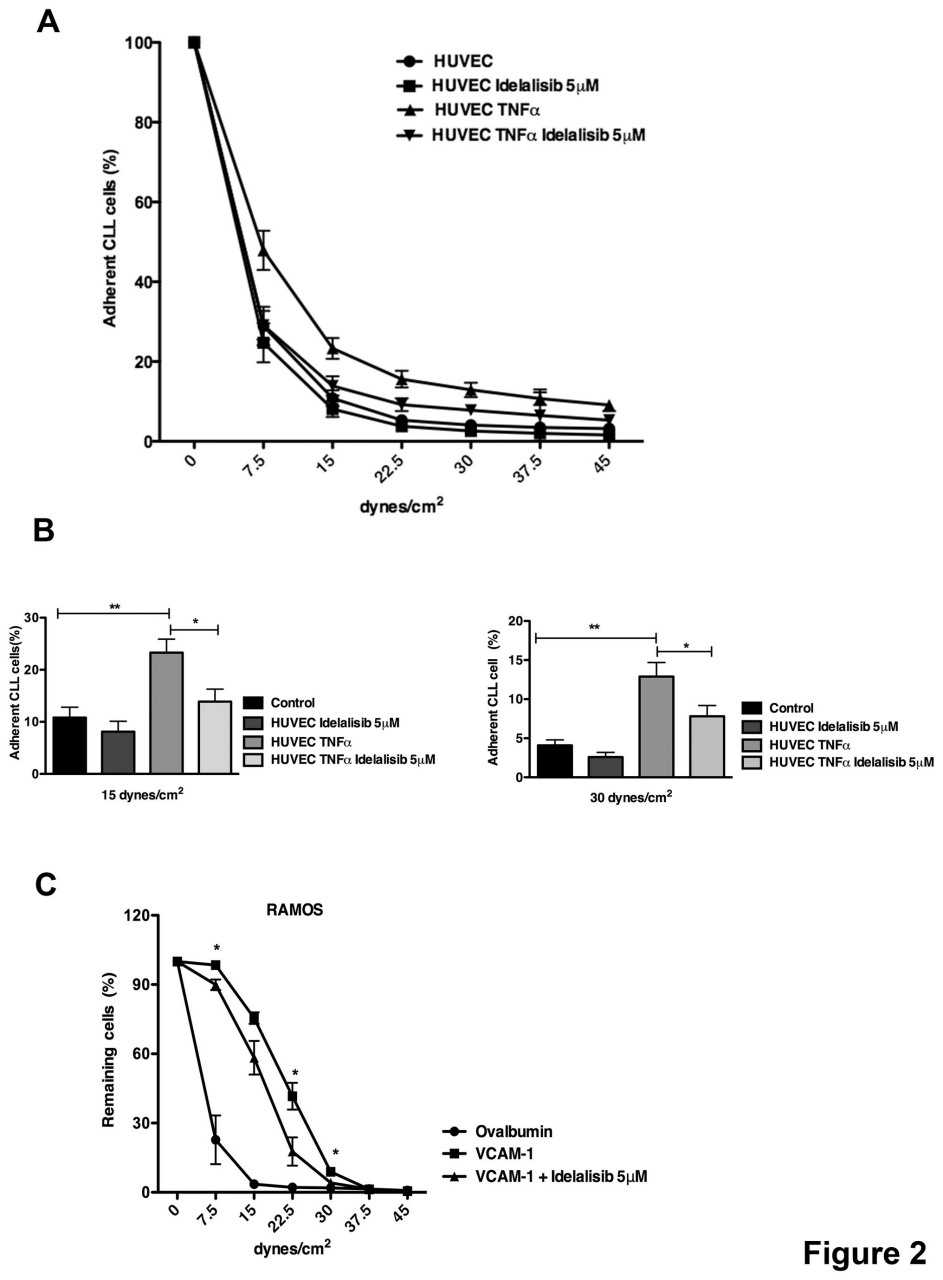
Adhesion to activated endothelium and VCAM-1 is inhibited by idelalisib. A linear gradient of shear flow increasing from 0-45 dynes/cm^2^ is applied over the adhered cells and the number of adherent cells is determined by the percentage of cells remaining every 50 seconds. (A) CLL cells were either treated or not treated with idelalisib and allowed to adhere to HUVEC stimulated or not stimulated (control) with TNFα (n=5). The data displayed the mean relative adhesion (±SEM) at different shear forces compared with the baseline adhesion for each condition. (B) The bar diagrams display the percentage of cells adherent to HUVEC at two representative shear forces (15 and 30 dynes/cm^2^). Displayed are the means (±SEM) from 5 different patients. idelalisib significantly inhibits the adhesion of CLL cells to HUVEC TNFα stimulated at low and high shear forces (*p<0.05; **p<0.01). (C) Ramos cells were treated or not treated with idelalisib and allowed to adhere to slides coated with VCAM-1 or ovalbumin (control). Idelalisib is able to interfere with VCAM-1-mediated adhesion at different shear forces (n=3, *p<0.05). The data display the mean relative adhesion (±SEM) at different shear forces compared with the baseline adhesion for each condition.

To investigate effects of idelalisib on CLL cell adhesion to EC under conditions that mimic physiologic flow conditions within blood vessels, we used parallel plate flow assays. CLL cells treated with 5μM idelalisib or unstimulated control cells were allowed to adhere to TNFα-stimulated HUVEC. As shown in Figure 2A, TNFα treatment increased CLL cell adhesion to HUVEC when compared with unstimulated controls. Idelalisib effectively inhibited CLL cell adhesion to EC under shear flow conditions at low (15 dynes/cm^2^) and higher shear forces (30 dynes/cm^2^). As illustrated in Figure 2B, the mean (±SEM) relative number of adherent CLL cells on HUVEC increased from 10.8 % (±2%) to 23.3 % (±2.6%) after TNFα stimulation (p<0.01, n=5) at 15 dynes/cm^2^. Idelalisib decreased CLL cell adhesion to TNFα-activated HUVEC from 23.3% (±2.6%) to 13.9% (±2.4%) at low shear force of 15 dynes/cm^2^ (p<0.05, n=5). At high shear force 30 dynes/cm^2^ the mean (±SEM) number of cells adherent to HUVEC increased from 4.1% (±0.7%) to 12.9 (±1.8%) in presence of TNFα stimulation (p<0.01, n=5) that decreased to 7.8% (±1.4%) in presence of 5μM idelalisib (p<0.05, n=5). 

As previously described, treatment with TNFα induces VCAM-1 expression on endothelial cells [34,35] and in addition, BCR activation increases adhesion to VCAM-1 of primary cells and B cell lines [36]. Therefore, we investigated specifically if adhesion mediated by VCAM-1 was affected by idelalisib in a B cell line. Ramos cells were treated and not treated with idelalisib and allowed to adhere to slides coated with VCAM-1. As shown in Figure 2C, idelalisib significantly inhibited Ramos cell adhesion to VCAM-1. At a shear force of 22.5 dynes/cm^2^ the mean (±SEM) number of cells adherent to VCAM-1 decreased from 41.6%±5.8% to 17.7%±6.1% in presence of idelalisib (n=3; p<0.05). Collectively, these data confirm the earlier results in the static adhesion assays, corroborating that idelalisib inhibits CLL cell adhesion to activated endothelium and in particular suggest an interference with the interaction between VLA-4 and VCAM-1.

### VLA-4 expression on CLL cells and VCAM-1 on EC/BMSC increase CLL adhesion, which is inhibited by idelalisib

**Figure 3:**
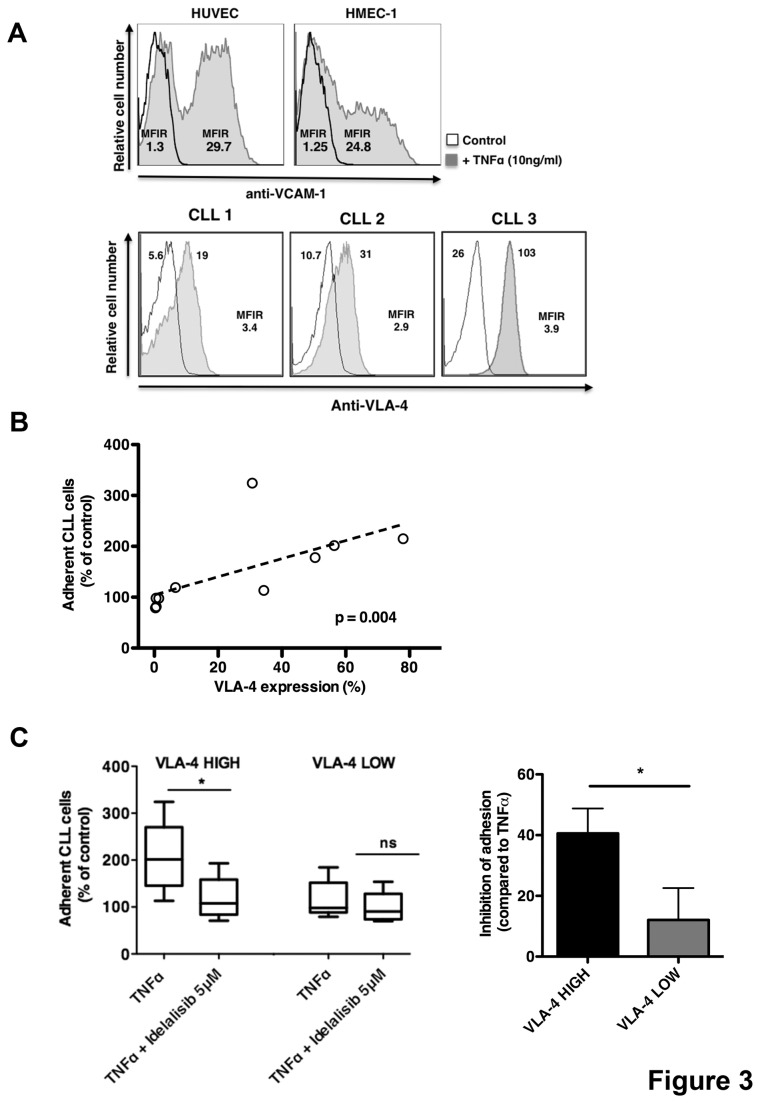
Idelalisib affects adhesion mediated by VLA-4 and VCAM-1 interaction. (A) On the left, displayed are fluorescence histograms depicting the relative fluorescence intensity of HUVEC and HMEC-1 stained with anti-VCAM-1 mAb either treated or not treated (control) with TNFα (10ng/ml) for 24 hours. Mean fluorescence intensity ratio was calculated by dividing the mean fluorescence intensity for VCAM-1 by the mean fluorescence of the isotype control in both conditions. On the right, representative fluorescence histograms of CLL cells stained with anti-VLA-4 (grey histograms) or the corresponding isotype control (white histograms). THE MFIR is displayed below the histograms. (B) A positive correlation is displayed between VLA-4 expression (%) and the relative adhesion of CLL cells to HUVEC stimulated with TNFα (n=10). (C) The box plot displays a comparison of CLL cell adhesion to HUVEC TNFα-stimulated in presence or absence of idelalisib in VLA-4 high (n=5) and VLA-4 low (n=5) expression groups. Bar diagram represents the percentage of inhibition in CLL adhesion to HUVEC stimulated with TNFα induced by idelalisib in VLA-4 high and VLA-4 low CLL samples relative to adhesion observed without idelalisib. Data are shown as mean ±SEM (*p<0.05).

Next, we addressed the effect of increased adhesion mediated by TNFα to more adhesive interactions between VLA-4 on CLL cell and VCAM-1 expressed on EC and BMSC. We analyzed VCAM-1 expression before and after TNFα treatment on EC and BMSC and VLA-4 in CLL cells. We confirmed that TNFα treatment strongly upregulated VCAM-1 expression on the surface of HUVEC and HMEC-1 (n=3), as shown in Figure 3A. Moreover CLL cells showed variable expression of VLA-4 integrin ranging from 1% to 78%, as shown in 3 positive representative samples (cut-off of 30%) in Figure 3A. Given the high expression of VCAM-1 on EC/BMSC and the variable VLA-4 expression on CLL cells, we compared the relative adhesion of CLL cell to activate endothelium with their individual VLA-4 expression. We observed a significant positive correlation between VLA-4 expression (**%**) and adhesion to TNFα-treated HUVEC (Figure 3B; r=0.818, p=0.004). These data were also confirmed using mean fluorescence intensity ratios for VLA-4 (data not shown). Next, we investigated the ability of idelalisib to interfere with CLL adhesion in VLA-4 high and VLA-4 low groups. The mean relative adhesion (±SEM) of CLL cells on TNFα-treated HUVEC layer significantly decreased from 206% (±34.2%) to 118.5% (±20.5%) (n=5, p<0.05) in VLA-4 high expression group, and from 115.6% (±18.3) to 98.8% (±14.8%) in VLA-4 low expression group (n=5, p=ns). Using idelalisib to interfere with VLA-4-VCAM-1 interaction, we found a strong inhibition of CLL adhesion to TNFα-treated endothelium in VLA-4 high expression group (40.6% inhibition of adhesion) compared with VLA-4 low expression group (12.1% of inhibition of adhesion) (Figure 3C, n=10, p<0.05). Collectively, our data suggest that idelalisib significantly inhibits CLL adhesion to EC and BMSC interfering with VLA-4 and VCAM-1 interaction.

### Idelalisib inhibits CLL cell survival in EC and BMSC co-culture

**Figure 4:**
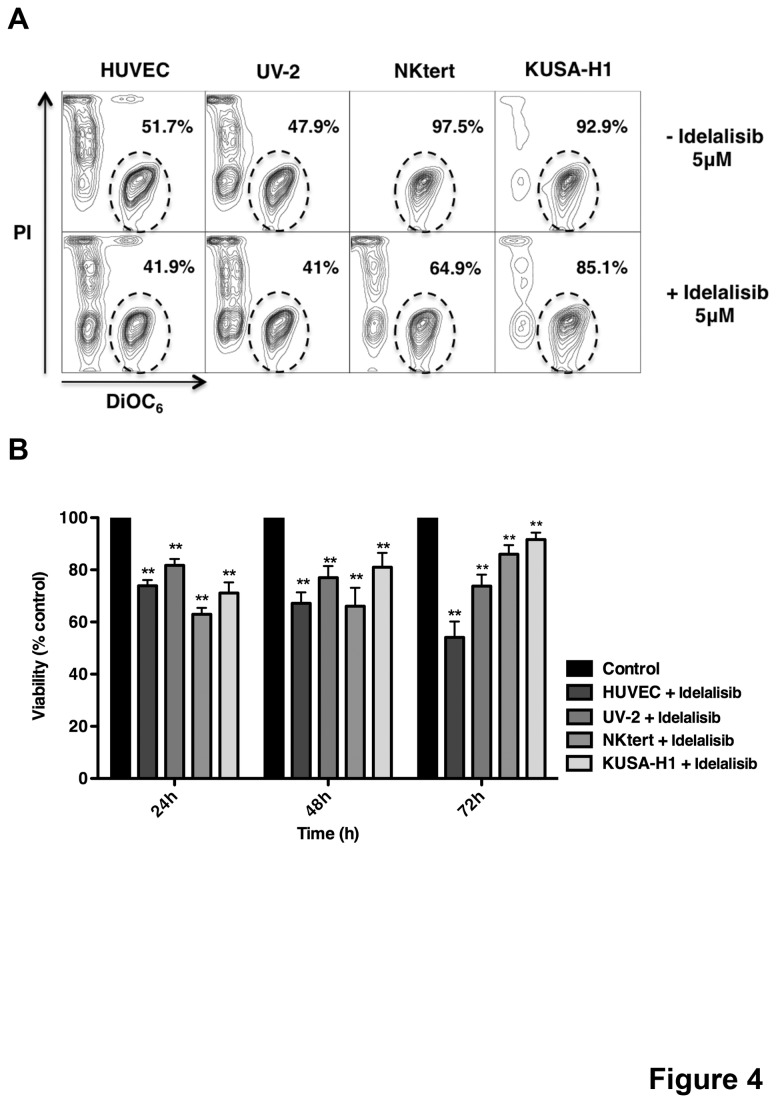
Idelalisib overcomes endothelial cells and bone marrow stromal cells mediated CLL cell protection. (A) CLL cells were co-cultured with HUVEC, UV-2, NKtert and KUSA-H1 in presence or absence of 5μM idelalisib. The gates in the contour plots exemplify viable CLL cells in one representative sample. (B) The bar diagrams represent the mean relative viabilities of CLL cells co-cultured in normal conditions (control) or in presence of 5μM idelalisib with EC and BMSC. Viabilities in idelalisib-treated samples were normalized to the corresponding viabilities of control samples at the respective time-point (100%) to account for differences in spontaneous apoptosis. Displayed is mean±SEM from 7 different samples (*p<0.05; **p<0.01).

BMSC and to a lesser degree EC are recognized for their capacity to protect CLL cells from spontaneous apoptosis in a contact-dependent fashion. To elucidate the relative capacity of BMSC versus EC for protecting CLL cells, we performed a direct comparison of these different support systems and measured effects of idelalisib in these co-cultures. While both, EC and BMSC support the viability of CLL cells, we noted comparably less protection from spontaneous apoptosis when compared directly with BMSC (Figure S1 A,B). To test the effect of idelalisib on EC- and BMSC- mediated CLL cell protection, CLL cells were cultured on EC (HUVEC and UV-2) or BMSC (NKtert and KUSA-H1) in presence or absence of 5μM idelalisib and CLL cell viabilities were assessed at 24, 48, 72 hours. First, we tested the effects of idelalisib on EC and BMSC and we found no influence on viability and morphology (data not shown). In a representative case (Figure 4A), idelalisib reduced CLL cell viability from 51.7% to 41.9% with HUVEC, 47.9% to 41% with UV-2, 97.5% to 64.9% with NK-tert and 92.9% to 85.1% with KUSA-H1 after 48 hours. We also found a significant reduction in the viability of CLL cells co-cultured with EC and BMSC in presence of idelalisib compared to the control at all time-points. At 24 hours, the mean relative viability of CLL cells in presence of idelalisib was 73.9% (±2.2%, p<0.01, n=7) with HUVEC, 81.7% (±2.5% p<0.01, n=7) with UV-2, 63.0% (±2.4% p<0.01, n=7) with NKtert and 71.1% (±4.1% p<0.01, n=7) with KUSA-H1 (Figure 4B). These data demonstrate that idelalisib can overcome EC- and BMSC- mediated CLL cell protection. In addition the impact of idelalisib is roughly equivalent in its ability to overcome CLL B cell protection for both cell types.

### Idelalisib inhibits EC- and VLA-4-induced signaling in CLL cells

**Figure 5:**
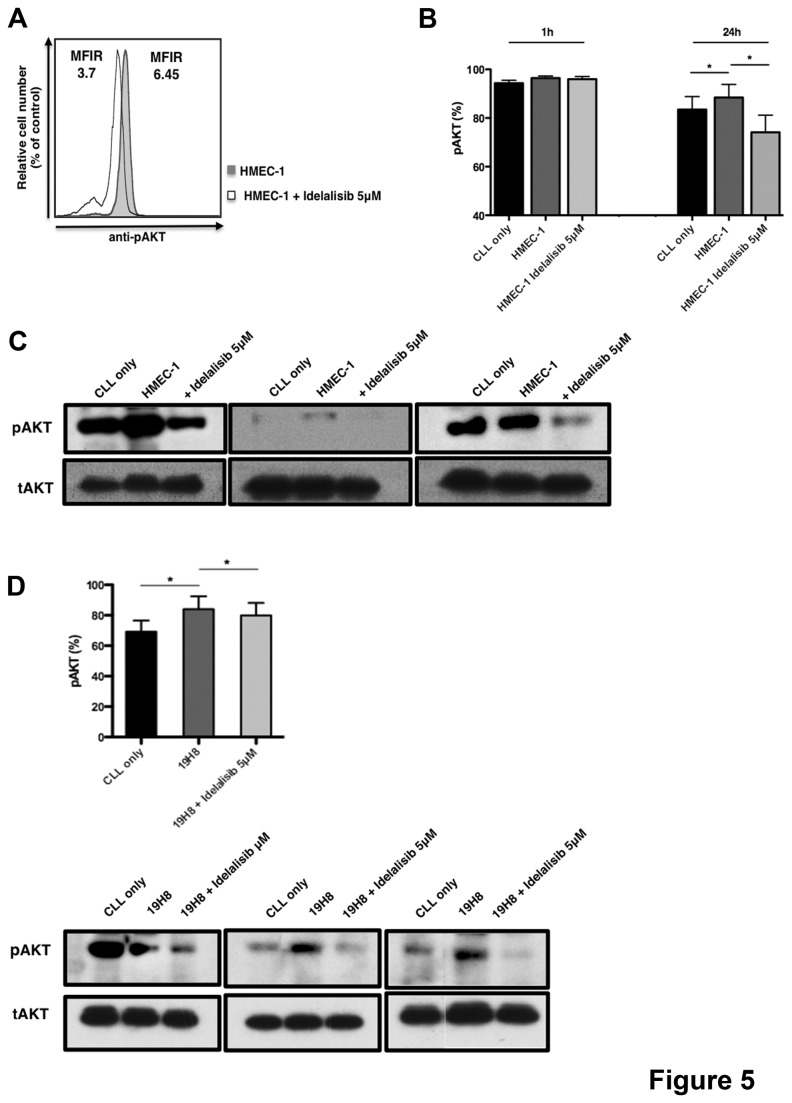
Idelalisib down-regulates integrin-induced AKT phosphorylation. (A) Overlay histograms depict the mean AKT phosphorylation (pAKT) fluorescence intensity of CD19+ CLL cells in a representative case. The solid grey histogram depicts pAKT staining of CLL cells in co-culture with HMEC-1 without idelalisib and the black line histogram depicts of CLL cells in co-culture with HMEC-1 in presence of 5 μM idelalisib. AKT phosphorylation was analyzed in CLL cells after 1 hour and 24 hours of co-culture with HMEC-1 either in presence or absence of idelalisib. pAKT was evaluated by flow cytometry in CD19^+^ CLL cells gated from 4 different patients. (B) CLL cells were cultured in medium alone and in co-culture with HMEC-1, either in presence or absence of idelalisib for 1 hour or 24 hours. The bar diagrams represent the mean ± SEM of pAKT positive cells (%) (n=4, p<0.05). (C) Displayed are immunoblots from 3 representative CLL cell samples cultured alone or in co-cultured with HMEC-1 in presence or absence of idelalisib for 24 hours. Lysates were probed with antibodies to pAKT (S473) and actin. (D) CLL cells were stimulated or no stimulated with anti-VLA4 mAbs (clone 19H8) in the presence or absence of idelalisib. Displayed are the means ± SEM from 5 different patients (*p<0.05). The immunoblots on the right hand side depict AKT activation in 3 representative CLL samples stimulated with anti-VLA4 in presence or absence of idelalisib.

To determine effects of idelalisib on EC-induced signaling, we analyzed AKT phosphorylation in presence or absence of idelalisib after co-culture with HMEC-1 by flow cytometry. Using mean fluorescence intensity ratios (MFIR), we quantified AKT phosphorylation and normalized for the MFIR of CLL cell cultured alone. Overlay histograms depict relative pAKT expression in a representative CLL sample co-cultured with HMEC-1 for 24 hours in presence or absence of idelalisib (Figure 5A). Figure S2A shows mean relative levels of pAKT, measured after 1 and 24 hours of EC co-culture. While differences after 1 hour of co-culture were small, AKT phosphorylation was increased after 24 hours in co-culture with HMEC-1 from 100.9% (±6.8%) to 117.2% (±7.6%) (p<0.01, n=4). Idelalisib significantly decreased EC-induced pAKT levels to 76.7% (±8.8%) after 24 hours (p < 0.05). We consistently found that after 24h, CLL adhesion to EC increased the percentage of CLL positive for AKT phosphorylation in CLL cells from 83.5% (± 5.3%) to 88.5% (5.4%) (n=4, p<0.05) and idelalisib decreased EC-induced AKT phosphorylation to 74.1% (±7.8%) (n=4; p<0.05) as shown in Figure 5B. These findings were confirmed by immunoblot analyses (Figure 5C and Figure S2B), which confirmed an increased AKT phosphorylation induced by CLL cell adhesion to EC and pAKT inhibition in response to idelalisib treatment. Furthermore we investigated, if VLA-4 activation can also induce AKT activation, therefore we incubated CLL cells with activating anti-VLA-4 (19H8) mAbs for 1 hour, followed by crosslinking in the presence or absence of idelalisib. As shown in Figure 5D, AKT phosphorylation induced by VLA-4 stimulation was able to increase AKT phosphorylation in CLL from 69.2% (±7.4%) to 84.1% (±8.4%) (n=5, p<0.05) and in presence of idelalisib pAKT decreased to 79.9% (±8.3%) (n=5, p<0.05). The crosslinking secondary antibody alone did not stimulate AKT phosphorylation (data not shown). This result was confirmed also by immunoblot with 3 different CLL samples (Figure 5D and Figure S3B). These data demonstrate that idelalisib is able to inhibit AKT phosphorylation induced by EC and VLA-4-triggering, indicating that idelalisib interferes with CLL cell adhesion by inhibition of VLA-4 integrin function and signaling.

## Discussion

Currently, several inhibitors of kinases downstream of B cell receptor are developed in clinical trials in CLL, and are emerging as promising alternative targeted therapies for CLL patients. These new agents include the spleen tyrosine kinase (Syk) inhibitor fostamatinib [11], Bruton’s tyrosine kinase (BTK) inhibitor ibrutinib (formerly PCI32765) [12], and the PI3Kδ inhibitor idelalisib (formerly CAL-101) [16], and all have shown similar characteristic clinical activity. Typically, these agents cause rapid resolution of lymphadenopathy and/or organomegaly with redistribution of CLL from tissues into the blood, resulting in a surge in lymphocytosis during the first weeks of therapy, which later oftentimes slowly resolves. This characteristic activity suggests that these agents interfere with the CLL cell adhesion and other tissue retention signals.

Our previous works on idelalisib [16], ibrutinib [37], and R406 [38] suggest that disruption of chemokine receptor signaling and function by these inhibitors is involved in the CLL cell redistribution phenomenon. Mechanistically, this concept, which is largely based on chemotaxis assays and signaling studies, likely does not fully explain the clinical findings given the plethora of means by which CLL B cells can adhere to stromal cells. To better characterize and understand the nature of the idelalisib-induced CLL cell redistribution, we explored the effects of idelalisib on CLL cell adhesion to different stromal cell types present in the CLL microenvironment (BMSC, EC). In our in vitro models, we analyzed CLL cell adhesion under static and shear flow conditions. Our results confirmed that interactions between VLA-4 (CD49d) on CLL cells and VCAM-1 (CD106) on the stromal cell surface play an important role in CLL cell adhesion to stromal cells [39]. Treatment of EC and BMSC with TNFα strongly increased VCAM-1 expression on the stromal cells that facilitated adhesion of CLL cells via VLA-4 expression. Our data demonstrate that idelalisib affects CLL adhesion to both, EC and BMSCs, which is more pronounced in CLL cells with higher VLA-4 expression, corroborating the importance of VLA-4 and VCAM-1 in CLL cell adhesion. Interestingly, and along the same lines, de Rooij and colleagues recently reported that the BTK inhibitor ibrutinib strongly inhibits VLA4 integrin-mediated adhesion of lymphoma cell lines and primary CLL cells to fibronectin and VCAM-1, and proposed this activity, along with inhibition of chemokine receptor function, as an underlying mechanism to explain the CLL cell redistribution caused by this BTK inhibitor [40]. Our data corroborate this concept and extend de Rooij’s findings in our CLL co-culture systems with both BMSC and EC. This is of relevance to the in vivo situation in that these cells will more closely resemble the in vivo microenvironment. In this scenario, CLL cells enter and accumulate into lymph nodes where adhere to accessory cells using different integrins and in particular VLA-4. This induces an increase in lymph nodes size supported by different growth and survival signals. Idelalisib is able to interfere with integrin-mediated adhesion, in particular with VLA-4 on CLL cells and VCAM-1 on EC/BMSC, causing lymph node shrinkage with a redistribution of CLL into the blood.

Besides tissue homing and retention, increased CLL cell survival is an expected consequence of adhesion to stromal cells, but effects of idelalisib on EC- and BMSC-mediated CLL cell survival have not previously been studied in detail. The novel findings in this study are the characterization of idelalisib effects on CLL cell adhesion to BMSC and EC under static and shear flow conditions, its effects on CLL cell viability in side-by-side co-cultures with BMSC and EC, and the role of the VLA-4-VCAM-1 axis in AKT pathway activation in CLL. More specifically, we found that BMSC were consistently more effective in maintaining CLL cell viability when compared with EC. The exact mechanism for this is unclear but seems not too surprising given that BMSC are critical and more numerous components of the supporting cellular infrastructure for hematopoietic cells in the marrow microenvironment. The finding that idelalisib can significantly interfere with these pro-survival effects implies that the adhesive interaction, and not exclusively BCR activation and signaling, contributes to CLL cell activation and survival in the tissue sites. Another new finding in this study is related to the specific stimulation of VLA-4 integrins on CLL cells causing increased the activation of the pro-survival AKT pathway in the process of CLL adhesion to EC or BMSC. Idelalisib inhibited AKT pathway activation, although to variable degrees in different patient samples, supporting a role of PI3Kδ in integrin signaling. 

In conclusion, our study demonstrates that idelalisib interferes with CLL cell adhesion to different cells present the tissue microenvironment by inhibiting interactions between VLA-4 on CLL cells and VCAM-1 on stromal and endothelial cells. Idelalisib not only disrupts CLL cells adhesion, but also thwarts pro-survival pathways activated during CLL cell adhesion. These data provide novel insight into the mechanism of action of idelalisib that relate to the redistribution of CLL cells during idelalisib therapy. 

## Supporting Information

Figure S1 **(**A**) CLL cells were cultured in medium alone, in co-culture with two EC (HUVEC and UV-2) and in co-culture with two BMSC (NKtert and KUSA-H1) for three different time-points: 24, 48, 72 hours.** Contour plots display a representative CLL sample viability after 48 hours. The gates represent the viable cells positive to DiOC_6_ and negative to PI. (B) The line graph displays mean±SEM CLL viabilities from 7 different patients after 24, 48, 72 hours of culture. Both EC and BMSC significantly support CLL viability at different time-points (*p<0.05; **p<0.01) compared to CLL culture in medium alone.
(DOC)

Figure S2 **(**A**) The bar diagrams represent the mean fluorescence intensity ratio (MFIR) for AKT phosphorylation in CLL cells co-cultured with HMEC-1, either treated or no treated with idelalisib, normalized for the MFIR of CLL cultured alone (control).** MFIR was calculated by dividing the mean fluorescence intensity for pAKT by the mean fluorescence of the respective isotype control. (B) Displayed are immunoblots from 2 representative CLL samples of 4 patients co-cultured with HMEC-1 in presence or absence of idelalisib for 24 hours. Lysates were probed with antibodies to pAKT (Tyr 308) and actin.
(DOC)

Figure S3 **A) The bar diagrams represent the mean relative fluorescence intensity ratio of CLL cells stimulated with 19H8 mAb (VLA-4) either in presence or absence of idelalisib.** Mean fluorescence intensity ratio were normalized for the corresponding MFIR at baseline. Displayed are the means (±SEM) from 3 different patients (*p<0.05; **p<0.01, n=3). B) The immunoblot depicts AKT activation (T308) in two representative CLL samples stimulated with 19H8 anti-VLA4 mAbs in presence or absence of idelalisib.
(DOC)
