# Supplementary figures and images for: The PI3-Kinase Delta Inhibitor Idelalisib (GS-1101) Targets Integrin-Mediated Adhesion of Chronic Lymphocytic Leukemia (CLL) Cell to Endothelial and Marrow Stromal Cells
Source: PLoS One. 2013 Dec 23;8(12):e83830. doi: 10.1371/journal.pone.0083830 (PMC3871531; doi:10.1371/journal.pone.0083830)

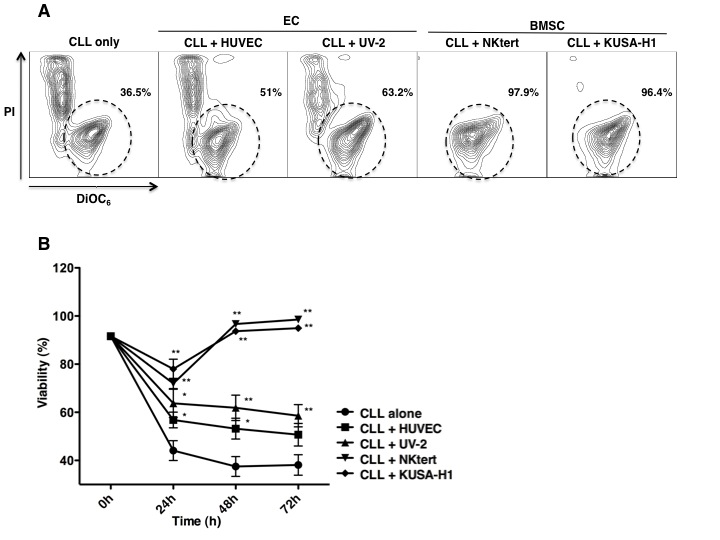


**Figure S1.**

Supplement: Figure S1 — (A) CLL cells were cultured in medium alone, in co-culture with two EC (HUVEC and UV-2) and in co-culture with two BMSC (NKtert and KUSA-H1) for three different time-points: 24, 48, 72 hours. Contour plots display a representative CLL sample viability after 48 hours. The gates represent the viable cells positive to DiOC6 and negative to PI. (B) The line graph displays mean±SEM CLL viabilities from 7 different patients after 24, 48, 72 hours of culture. Both EC and BMSC significantly support CLL viability at different time-points (*p<0.05; **p<0.01) compared to CLL culture in medium alone. (DOC) [file pone.0083830.s001.doc]

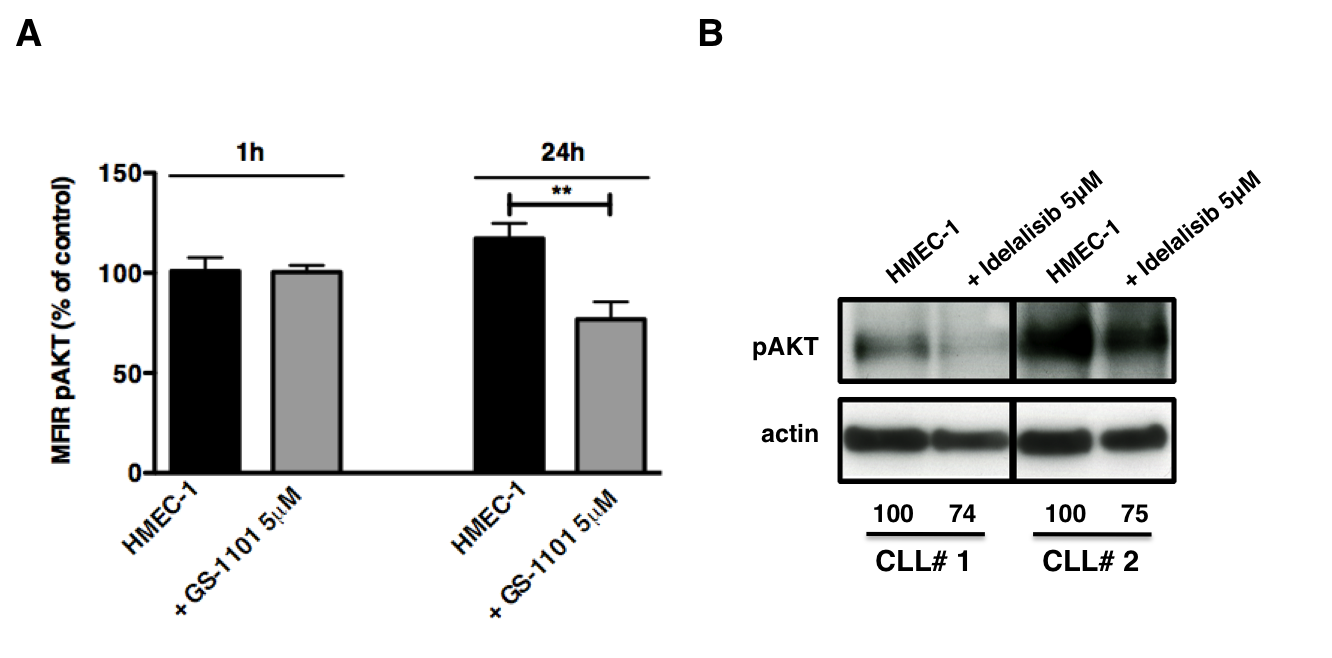


**Figure S2.**

Supplement: Figure S2 — (A) The bar diagrams represent the mean fluorescence intensity ratio (MFIR) for AKT phosphorylation in CLL cells co-cultured with HMEC-1, either treated or no treated with idelalisib, normalized for the MFIR of CLL cultured alone (control). MFIR was calculated by dividing the mean fluorescence intensity for pAKT by the mean fluorescence of the respective isotype control. (B) Displayed are immunoblots from 2 representative CLL samples of 4 patients co-cultured with HMEC-1 in presence or absence of idelalisib for 24 hours. Lysates were probed with antibodies to pAKT (Tyr 308) and actin. (DOC) [file pone.0083830.s002.doc]

**Figure S3.**


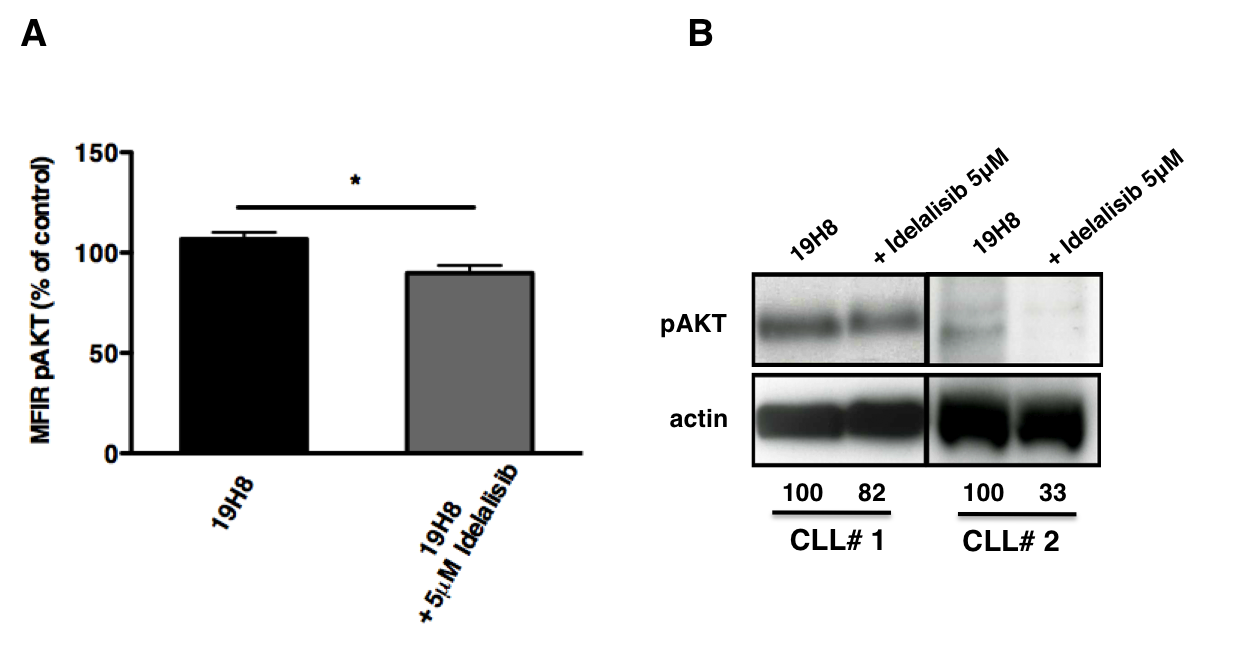

Supplement: Figure S3 — A) The bar diagrams represent the mean relative fluorescence intensity ratio of CLL cells stimulated with 19H8 mAb (VLA-4) either in presence or absence of idelalisib. Mean fluorescence intensity ratio were normalized for the corresponding MFIR at baseline. Displayed are the means (±SEM) from 3 different patients (*p<0.05; **p<0.01, n=3). B) The immunoblot depicts AKT activation (T308) in two representative CLL samples stimulated with 19H8 anti-VLA4 mAbs in presence or absence of idelalisib. (DOC) [file pone.0083830.s003.doc]
